# Precision Medicine in Hormone Receptor-Positive Breast Cancer

**DOI:** 10.3389/fonc.2018.00144

**Published:** 2018-05-04

**Authors:** Azadeh Nasrazadani, Roby A. Thomas, Steffi Oesterreich, Adrian V. Lee

**Affiliations:** ^1^Department of Medicine, University of Pittsburgh, UPMC Hillman Cancer Center, Pittsburgh, PA, United States; ^2^Women’s Cancer Research Center, Department of Pharmacology and Chemical Biology, UPMC Hillman Cancer Center, Magee Womens Research Institute, Pittsburgh, PA, United States

**Keywords:** hormone receptor-positive breast cancer, personalized medicine, liquid biopsy, circulating tumor DNA, circulating tumor cells

## Abstract

In recent decades, breast cancer has become largely manageable due to successes with hormone receptor targeting. Hormone receptor-positive tumors have favorable outcomes in comparison to estrogen receptor (ESR1, ER)/progesterone receptor-negative tumors given the targetable nature of these tumors, as well as their inherently less aggressive character. Nonetheless, treatment resistance is frequently encountered due to a variety of mechanisms, including *ESR1* mutations and loss of ER expression. A new era of precision medicine utilizes a range of methodologies to allow real-time analysis of individual genomic signatures in metastases and liquid biopsies with the goal of finding clinically actionable targets. Preliminary studies have shown improved progression-free survival and overall survival with implementation of this information for clinical decision making. In this review, we will discuss the opportunities and challenges in integrating precision medicine through next-generation genomic sequencing into the management of breast cancer.

## Hormone Receptor-Positive Disease

Breast cancer is the most common cancer in US women, and the second leading cause of death. It is estimated that 252,710 women in the US will be diagnosed with invasive breast cancer this year, and 40,610 women will succumb to this disease during this time. Despite these disquieting statistics, relative survival rates are quite high, estimated to be 91% at 5 years post diagnosis and 80% at 15 years (1, 2). In large part excellent outcomes can be attributed to successes with targeting hormone receptors in hormone receptor-positive disease, which comprises 83% of invasive breast cancers according to recent data (1).

Hormone receptor inhibition has been pursued as far back as the 1960s, albeit initially with the goal of developing contraceptives. Tamoxifen, initially known as ICI 46474, was considered a failure when it was found to stimulate rather than suppress ovulation while being investigated for its intended use as an anti-estrogen (3). It was not until the 1970s that it was analyzed for its antitumor effects, obtaining approval for use in advanced breast cancer in 1977. Over time, tamoxifen would gain wide acceptance after studies demonstrated a clear reduction in mortality with use in the adjuvant setting (4–7). Meta-analyses of 15 years follow-up data from randomized trials of patients with ER-positive disease given 5-year adjuvant tamoxifen after 6 months of anthracycline-based chemotherapy have demonstrated an approximately 50% reduction in breast cancer mortality (8).

Gene expression signatures based on microarray studies have distinguished breast cancer into various molecular intrinsic subtypes (luminal A, luminal B, *HER2*-like, basal-like, and normal-like); however, hormone receptor positivity remains the fundamental feature of this disease (9, 10). ER/progesterone receptor (PR) status, specifically, drives the recommendation to initiate hormone targeted therapies, defined as the presence of at least 1% positive staining in tumor nuclei *via* immunohistochemistry (IHC) testing (11, 12). Standard of care dictates endocrine therapy for patients with ER-positive disease, regardless of other parameters, as per guidelines by ASCO, ESMO, and St. Gallen International Expert Consensus (13–15).

ER expression serves not only as a predictive biomarker for response to endocrine therapy but also provides prognostic value. ER positivity has been found to confer a survival advantage in patients treated with endocrine therapy, specifically tamoxifen, which has not been observed with ER negative disease (5, 16–18). Several reports have documented amplification of estrogen receptor 1 (*ESR1*), although this remains controversial. Interestingly, *ESR1* amplification has not been found to correlate with outcomes in it of itself, although an ER-related transcription signature developed by Symmans et al., termed a sensitivity to endocrine therapy index, demonstrated a significant association with distant relapse-free survival (19).

Tamoxifen is a selective estrogen receptor modulator, exerting inhibitory effects on estrogen signaling by functioning as a competitive inhibitor, promoting ER conformational change, which prevents coactivator binding and thereby hindering propagation of downstream signaling (20). Tamoxifen has been the first-line agent of choice for use in hormone receptor-positive disease for many years. Interestingly, two genetic events that alter generation of estradiol and metabolism of tamoxifen may be important in breast cancer. A recent report indicated that *CYP19A1*, the aromatase gene responsible for generation of estradiol in postmenopausal women, is amplified in endocrine-resistant breast cancer (21). Tamoxifen is metabolized by *CYP2D6* to endoxifen, and evidence suggests that certain CYP2D6 genotypes have reduced metabolism and level of endoxifen, which may be associated with worse outcomes (22). However, this is a controversial area with several studies not supporting this (23). Most recently, the tamoxifen metabolite, endoxifen, has shown promise in a phase I study in women with endocrine refractory metastatic disease, demonstrating a clinical benefit rate or partial response of 26% (24).

Evidence now supports upfront use of aromatase inhibitors (AIs), specifically in the postmenopausal population. AIs block the synthesis of estrogen from non-ovarian, precursor steroids, and have demonstrated superiority with regard to overall response rates, progression-free survival (PFS), and 10-year mortality rates in comparison to tamoxifen in this population (25, 26). The lower incidence of thromboembolic events and vaginal bleeding with AIs further support their use over tamoxifen (27–29).

Selective estrogen receptor degraders (SERDs) encompass an emerging class of hormone receptor-targeted therapies with fulvestrant currently as the only approved agent to date. Fulvestrant inhibits ER dimerization, reducing its nuclear translocation, leading to accelerated receptor degradation and ultimately resulting in the complete suppression of the estrogenic effects on breast tissue (30). Results of the phase III FALCON study comparing use of fulvestrant to anastrozole in advanced hormone receptor-positive breast cancer suggest fulvestrant could be considered in the first-line setting given its noted improvement in PFS (hazard ratio 0.797; median PFS, 16.6 versus 13.8 months, respectively) (31).

Despite tremendous advances in the treatment of hormone receptor-positive breast cancer, resistance remains a critical issue. The Early Breast Cancer Trialists’ Collaborative Group recently reported a meta-analysis of 20-year follow-up of 88 clinical trials involving 62,923 women with ER-positive breast cancer treated for 5 years with endocrine therapy (mainly tamoxifen). Importantly, breast cancer recurrences occurred at a steady rate throughout the period of 5–20 years (32). The multiple molecular mechanisms of resistance include alteration of ER expression, dysregulation of co-regulators, and cross talk with growth factor signaling pathways (33).

## Her2 (ERRB2)-Positive Breast Cancer

HER2 expression serves as a separate yet equally important parameter guiding breast cancer management and is also one of the key mediators of endocrine resistance (33). *HER2*, initially named *neu*, was identified as an oncogene as it was found to be activated in ethylnitrosourea-induced rat neuroblastomas (34). It was isolated by multiple groups based on its high homology with *v-erbB* and *EGFR*, specifically in the ATP-binding domain, but notably found to be distinct from *EGFR* itself (35–38). HER2 overexpression is demonstrated in approximately one quarter of breast cancers (39), with gene amplification of 2- to 20-fold estimated to occur in 30% of all breast tumors (40). *HER2* gene amplification confers a worse prognosis with shorter time to relapse and a decline in overall survival (OS), correlating with the degree of gene amplification (40, 41). Monoclonal antibodies targeting the extracellular domain of HER2 raised in cell lines expressing high HER2 levels showed promise in their ability to inhibit tumor cell proliferation *in vitro* (42–44) leading to eventual development of a humanized form (45) currently in use as the approved agent trastuzumab.

In patients with HER2-positive metastatic breast cancer (MBC), trastuzumab in combination with standard chemotherapy demonstrated a clear benefit in time to progression, duration of response, and OS as compared to chemotherapy alone (46). A notable 37 and 40% relative improvement in OS and DFS, respectively, would be demonstrated in women with surgically removed high risk disease receiving trastuzumab as part of standard therapy leading to eventual FDA approval in 2006 (47). Recurrence in this population, however, is not uncommon, prompting development of a range of agents targeting HER2 *via* distinct mechanisms. Pertuzumab is a humanized monoclonal antibody, which binds the extracellular domain of HER2 as trastuzumab, but at a separate epitope, thereby inhibiting receptor dimerization and preventing downstream signaling (48). The addition of pertuzumab to trastuzumab plus docetaxel in patients with HER2-positive MBC did demonstrate improved median survival by roughly 16 months (49). Lapatinib, an oral tyrosine kinase inhibitor to both epidermal growth factor receptor and HER2 was approved in combination with capecitabine for patients who had prior treatment with Trastuzumab refractory HER2-positive MBC (50). Other HER2-targeted modalities are notable for the development of ado-trastuzumab emtansine (T-DM1), an antibody drug conjugated to a cytotoxic agent (DM1). TD-M1 first gained approval when compared to lapatinib with capecitabine in the phase III EMILIA trial with an OS benefit of nearly 6 months (51). Single agent T-DM1 has been shown to significantly increase OS in patients with advanced disease with progression on multiple HER2-targeted therapies per findings from the TH3RESA trial (52). Another tyrosine kinase inhibitor that targets pan-HER2 receptors, neratinib, has recently gained approval in the extended adjuvant setting for patients with HER2-positive localized breast cancer based on the ExteNET study (53). Most recently, MYL-14010, a trastuzumab biosimilar developed for the purpose of improving global access and affordability was approved after it was demonstrated to be equivalent to trastuzumab with regard to outcomes and safety parameters (54).

## Targeting Other Pathways in Hormone Receptor-Positive Breast Cancer

The mammalian target of rapamycin (mTOR) pathway has a documented role in ER-resistant disease, with mTOR being of particular interest given its downstream position from the vital PI3K/AKT and Ras/Raf/Mek/Erk signaling pathways [reviewed in Ref. (55)]. Everolimus, a macrolide immunosuppressant and an mTOR inhibitor, has been utilized in treatment of various malignancies. The phase III BOLERO-2 trial demonstrated that everolimus in combination with exemestane, a steroidal AI, showed a median PFS of 7.8 versus 3.2 months for exemestane plus placebo based on investigator assessment (HR 0.45; 95% CI: 0.38–0.54; *P* < 0.0001), leading to FDA approval.

In an effort to target common dysregulated pathways in an otherwise highly heterogeneous disease, CDK4/6 inhibitors have come into favor in the recent years given their central role in cell cycle progression and interaction with ER. In an unchecked state in the setting of molecular aberrancy, uncontrolled transcription and proliferation ensues [reviewed in Ref. (56)]. Palbociclib, the first approved of the CDK4/6 inhibitors currently in use, is thought to preferentially inhibit growth in ER^+^ disease based on *in vitro* findings, which importantly demonstrated enhanced sensitivity in ER-resistant cell lines (57). In phase III PALOMA-2 and PALOMA-3 trials, Palbociclib in combination with an AI or SERD, respectively, demonstrated improvement with regard to PFS but not OS thus far in patients with advanced HR^+^ Her2^−^ disease (58, 59). Based on these studies, current guidelines advocate for concurrent use of palbociclib with hormone therapy in the first-line setting in patients with metastatic hormone-positive, HER2-negative disease. Subsequent FDA-approved CDK4/6 inhibitors have been similarly found to improve PFS when combined with an AI in the advanced setting. At an 18-month follow-up, HR^+^ Her2^−^ patients treated with Ribociclib plus letrozole had a 63% PFS rate, as compared to 42% in the placebo plus letrozole-treated population (60). Abemaciclib was first studied as a monotherapy in MBC patients with progression on prior lines of therapy, and demonstrated an overall response rate of 19*.7*% with a median PFS of 6 months, and median OS of 17.7 months (61). In the follow-up MONARCH 2 and MONARCH 3 trials, combination of abemaciclib with either fulvestrant or an AI, respectively, has consistently demonstrated an improved PFS when used in the first-line setting in patients with HR^+^ Her2^−^ advanced disease (62, 63). Most recently, a meta-analysis including the aforementioned CDK4/6 studies has confirmed the noted improvement in PFS and overall response rate when used in combination with an AI as first-line therapy in HR^+^ Her2^−^ patients with advanced breast cancer (64). Data are still being collected in relation to the utilization of rapamycin post-CDK4/6 therapy (65).

## Molecular Evolution and Endocrine Resistance in Hormone Receptor-Positive Disease

Recent evidence suggests that breast cancers evolve in response to various pressures including those such as lack of nutrients and oxygen, as well as in response to the application of targeted therapies. Part of this evolution is reflected by changes in expression of ER, PR, and HER2, with a meta-analysis indicating that these biomarkers change in approximately 20% of cases (66). Given this, ASCO recently changed guidelines to indicate that biomarkers should be measured on metastatic tissue if it is available, and therapy can be directed to that measured biomarker (67). Cejalvo et al. recently measured PAM50 intrinsic subtypes in 123 patient-matched pairs of primary and MBC (68). They found no changes in subtype in basal-like tumors, but changes in subtypes in HER2-enriched (23.1%), luminal B (30.0%), and in luminal A (55.3%) breast cancers. Luminal A primary breast cancers often converted to luminal B upon metastasis, and occasionally to from luminal to HER2-enriched. We recently reported similar changes in intrinsic subtypes switching in patient-matched pairs of primary and brain (69) and bone (70) metastases. A challenge of many of these studies is understanding the role of therapy in causing changes in biomarkers, yet a recent study sequencing primary and MBC in the *de novo* metastatic therapy naïve setting showed mutational differences (71).

Large international consortia have characterized the mutational landscape of primary breast cancer, and these studies have laid the foundation for detection and targeted therapies against mutations. However, the landscape in MBC remains understudied. Foundation Medicine reported on clinical genomic testing of all coding exons of 287 cancer-related genes plus select introns from 19 genes frequently rearranged in cancer in 18,004 cancers, with a large fraction being advanced cancers (72). They and other groups noted changes in frequency of mutations in MBC including increased mutation of *ESR1* (73–77). Specifically, the Y537S and D538G mutations have been found to confer shortened OS (78). These mutations are in the ligand-binding domain (LBD) and cause ER to exhibit ligand-independent activity. We recently reported recurrent *ESR1* gene fusions, where the LBD is removed and the ER becomes constitutively active, in endocrine-resistant breast cancer (79). Several reports have indicated amplification of *ESR1* (80), and we recently reported on response to high dose estradiol in a breast cancer with *ESR1* amplification (81).

## Monitoring Molecular Evolution and Resistance in ER-Positive Breast Cancer

Tissue collection becomes a particularly more complicated matter after resection of the primary tumor. Beyond issues with accessibility of metastatic sites, the need to obtain genomic information that is also temporally more reflective of the current state of the tumor has promoted the concept of liquid biopsies to gain favor. Liquid biopsies, which include the collection of circulating tumor cells (CTCs) or cell-free DNA (cfDNA) present in plasma, offer a relatively noninvasive method of monitoring disease, and in many cases, mutational status. CTCs, initially described as far back as 1869 in a postmortem case (82), are generally isolated based on EpCAM expression, and more recently also on physical properties of the CTCs. Cell Search is currently the only system with FDA approval for use in MBC. Methods in development are currently being pursued that instead rely on microfilters and the comparatively larger size of CTCs (83). CTC detection is challenging owing to extremely low frequency in circulation; however, multiple trials have been able to reliably demonstrate presence of CTCs in early and advanced breast cancer. These studies have consistently reported a worse prognosis with regard to PFS and OS with increase in the number of CTCs (84–88).

Cell-free DNA is easier to isolate than CTCs, requiring only a simple isolation of plasma and then DNA extraction. Circulating tumor DNA (ctDNA) can be identified in cfDNA by the presence of mutations that should not be present in normal germline DNA (generally isolated from white blood cells in buffy coat), and can be detected by an ever expanding number of techniques including digital droplet polymerase chain reaction (ddPCR) (89), BEAMing (beads, emulsion, amplification, magnetics) (90, 91), massively parallel sequencing (92), tagged amplicon deep sequencing (93), and the pyrophosphorolysis-activated polymerization method (94, 95). Early studies in cell-free tumor DNA present in serum revealed a significantly higher concentration in serum of patients with advanced disease as compared to patients without cancer or with slow growing tumors. Follow-up studies demonstrating decreased strand stability suggested the origin of this DNA is from tumor versus from normal tissue (96). There is debate regarding the exact mechanism of DNA release in plasma, although leading theories support spontaneous release versus apoptosis, and less likely release due to cell lysis or necrosis (97–99). Most recently, studies suggest cfDNA secretion is likely an active process that may occur in association with a protein complex (100). The feasibility of using these techniques for ctDNA detection and subsequent mutational analysis has been verified in multiple tumor types (101–103). In a recent study, analysis of alteration burden in ctDNA was found to predict response in patients with various malignancies receiving checkpoint inhibitors, indicating a more favorable outcome in OS and PFS in tumors with high alteration burden (104). In breast cancer, specifically, serial monitoring of ctDNA has been shown to predict disease recurrence months before metastasis could be clinically detected (105, 106), as has been found to be inversely correlated with OS (107).

Progression of disease or recurrence indicate either selection for clones resistant to treatment or evolution of the primary tumor with accumulation of mutations subsequently leading to therapy resistance. In either case, identification and sequencing of culprit mutations provide both a mechanism to predict response to therapies and outcomes, as well as a target to which future therapies can be developed. ddPCR and next-generation sequencing have been utilized by multiple groups to detect *ESR1* hotspot mutations in patients with advanced breast cancer (108) in an appreciable frequency (109–111), and higher in the metastatic setting as compared to micrometastatic disease (109, 112, 113). Not surprisingly, *ESR1* mutations have been demonstrated in patients with ER-positive disease previously exposed to AI therapy and found to confer a shorter PFS with subsequent AI therapy. The incidence of *ESR1* mutations were notably more frequent when exposed in the metastatic setting versus during adjuvant AI therapy (113).

## Guidance

Numerous groups are currently documenting molecular evolution in hormone receptor-positive disease, and current evidence indicates that *ESR1* is mutated (base pair, amplification, and fusion) in up to 50% of advanced endocrine-resistant breast cancers. However, the effect of these mutations on prognosis and response to endocrine therapy is unknown and thus the clinical actions to be taken upon the finding of an *ESR1* mutation are currently under intense investigation. Outside of *ESR1*, advanced endocrine-resistant breast cancers harbor mutations in many other pathways, but again the effect of targeting these mutations is currently under investigation. Investigators at Memorial Sloan Kettering Cancer Center recently reported on clinical genomic sequencing of over 10,000 advanced cancers and linked these to the OncoKB database of clinically actionable variants to show which ones have clinical utility (114). They were able to place 11% of patients on genomic-directed therapy. Lefebvre et al. reported not only on whole-exome sequencing of 216 advanced breast cancers and noted mutation and amplification of *ESR1*, but also mutation of clinically actionable genes *ERBB4, NOTCH3*, and *ALK* (115). Thoughtful agent selection becomes imperative, especially in the metastatic setting when time is more limited. Von Hoff et al. demonstrated an improvement in PFS in a range of refractory metastatic cancers treated with targeted therapies selected based on individualized FISH, microarray, or IHC data (116).

Despite the vast potential for liquid biopsy incorporation in clinical decision making, this field remains in its infancy. Numerous trials are currently underway evaluating the role of plasma ctDNA in both observational capacities to predict response, as well as from interventional perspectives [reviewed in Ref. (117)]. A Precision Oncology Decision Support System has been established at MD Anderson Cancer Center that provides a clinical interpretation service that comments on the clinical significance and actionability of alterations present in a tumor sample (118). Following the results of their recommendations, a small yet clinically significant improvement in OS and DFS was reported in the population in which the genomic panel annotations led to a change in therapy (119). A similar model can be envisioned with the use of liquid biopsies to guide selection of therapy as patients progress on their current treatment. Several trials are ongoing and in development, notably IMAGE and IMAGE-II studies initiated at the Sidney Kimmel Comprehensive Cancer Center at John Hopkins, which aim to identify actionable mutations in MBC as detected in tissue and blood.

Further studies are required to ensure what is gained in convenience in sample collection does not come at the expense of genomic expression concordance between tumor tissue and what is observed in liquid biopsy samples. Intra-heterogeneity demonstrated within individual CTCs from the same patient (120) brings into question the appropriateness of reliance on genomic data obtained from single cells. Furthermore, the limited half-life of circulating nucleic acids in circulation, estimated to be in the range of minutes (121), may greatly influence our ability to detect clinically significant alterations in a timely fashion.

At present, ASCO guidelines strongly recommend against use of data obtained from CTCs to guide decision making with regard to adjuvant systemic therapy (122) and cite a lack of evidence to suggest benefit in the metastatic setting (67). At least with regard to presence of *ESR1* mutations, however, Fribbens et al. report an improvement in PFS in patients with *ESR1* mutated, HR^+^, HER2^−^ MBC treated with fulvestrant as opposed to exemestane (110).

Current efforts in data collection using liquid biopsies in parallel with evidence-driven recommendations will indisputably provide a wealth of information that will continue to guide the field. As our understanding evolves by way of dynamic monitoring, the goal of utilizing liquid biopsies can approach an eventual reality. In a more real sense, however, an accurate reflection of a tumor profile will likely require incorporation of sources such as both cfDNA and CTCs, as well as RNA-based testing.

## Author Contributions

All authors participated in literature review and preparation of the manuscript.

## Conflict of Interest Statement

The authors declare that the research was conducted in the absence of any commercial or financial relationships that could be construed as a potential conflict of interest.
